# Enhanced Photocatalytic Removal of Hexavalent Chromium over Bi_12_TiO_20_/RGO Polyhedral Microstructure Photocatalysts

**DOI:** 10.3390/nano12132138

**Published:** 2022-06-22

**Authors:** Huihui Gan, Shuo Pan, Xiuhang Liu, Ying Huang

**Affiliations:** Civil and Environmental Engineering Department, Ningbo University, Ningbo 315100, China; panshuo86@163.com (S.P.); liuxiuhang7@163.com (X.L.); huangying1@nbu.edu.cn (Y.H.)

**Keywords:** bismuth titanate, reduced graphene oxide, photocatalytic, heavy metal pollutants

## Abstract

A Bi_12_TiO_20_/RGO photocatalyst with polyhedron microstructure was fabricated via the template-free hydrothermal method, and the visible-light-induced photocatalytic activity of the prepared Bi_12_TiO_20_ was also evaluated by the photocatalytic reduction of heavy metal pollutants. The structures and optical properties of the prepared Bi_12_TiO_20_/RGO were characterized by X-ray diffraction (XRD), scanning electron microscopy (SEM), and UV–vis diffuse reflectance spectrum (UV–vis DRS). The effects of the reaction time and mineralizer concentration on the formation of the Bi_12_TiO_20_ polyhedral microstructure were analyzed. The enhanced photocatalytic performances of Bi_12_TiO_20_/RGO were observed which were ascribed to the combination of the Bi_12_TiO_20_ microstructure induced photogenerated charges and the RGO nanostructure as a photogenerated charges carrier. The effect of organic acids, p-hydroxybenzoic acid (PHBA), chloroacetic acid, and citric acid on the Cr(VI) photocatalytic reduction was also discussed. This work provides an insight into the design of the bismuth-based microstructure photocatalyst towards the application for environment purification of heavy metals.

## 1. Introduction

Chromium is essential in many industrial activities, such as leather tanning, dyeing, glass, galvanic industries, and ceramics; however, it results in hexavalent chromium species in the wastewater. Unlike most organic pollutants, the heavy metal chromium is not biodegradable and its high mobility and toxicity in water pose a threat to the environment. Chromium existing as a hexavalent in water is listed as a priority pollutant in many countries due to its carcinogenic and mutagenic effect [1,2]. The traditional treatment methods, including adsorption, membrane filtration, biological treatment, chemical precipitation, and electrochemical reduction still have many drawbacks, such as having high energy consumption and being time-consuming [3]. Compared with Cr(VI), Cr(III) is much less toxic and mobile in water. Thus, the remediation reduction of Cr(VI) to Cr(III) has gained considerable attention. Recently, photocatalysis driven by solar energy for reducing Cr(VI) to Cr(III) has been the focus of numerous studies [4,5,6,7,8]. TiO_2_-WO_3_-PANI membranes were designed to realize the effective reduction of chromium (VI) in a reactor under visible light [9]. BiSI nanorods with rich sulfur vacancies were reported to be favorable for Cr(VI) adsorption and showed excellent photocatalytic Cr(VI) reduction ability [10]. In these methods, the photocatalytic reduction of Cr(VI) happens based on the photo-exited electron from the conduction band of photocatalysts or the generated reactive radical during the process under UV or visible light.

Bismuth-based materials have been considered as one of the alternative photocatalysts to TiO_2_ because of their unique electronic structures and visible-light response. Among bismuth oxides semiconductors, bismuth titanate Bi_12_TiO_20_ with its unique sillenite structure is a promising photocatalyst for the removal of organic pollutants owing to the excellent chemical stability, layered structure, and narrow optical bandgap. Moreover, it has been found that the properties of the photocatalysts can be further enhanced by controlling the synthesis of crystals with a fabricated morphology and an exposed highly active crystal facets [11,12]. Nanowires Bi_12_TiO_20_ were synthesized using titanium isopropoxide (TTIP) as starting materials and polyvinyl alcohol (PVA) as a surfactant, which displayed significantly improved activity for the photocatalytic decomposition of methyl orange under UV-light irradiation [13]. Flower-like Bi_12_TiO_20_ prepared by solvothermal treatment with TTIP was reported to fabricate Bi_12_TiO_20_–graphene nanoarchitectures and exhibited excellent photocatalytic activity for the degradation of methyl orange (MO) and p-nitrophenol (PNP) under simulated sunlight [14]. The Bi_12_TiO_20_ micro-tetrahedrons exposed by {111} facets coupled with g-C_3_N_4_ showed higher visible-light photocatalytic activity for degradation of gaseous formaldehyde [15]. Based on the above consideration, the fabrication of the Bi_12_TiO_20_ microstructure presents great appeal and expectation for promoting their photoreduction ability for Cr(VI).

Constructing an efficient photoinduced charge transfer interface is also critical for the higher efficient photocatalysts. Reduced graphene oxide (RGO) has been extensively researched and it has been clearly evidenced that assembling and decorating carbon nanosheets with the semiconductor can promote the photocatalytic and photoelectrochemical processes [16,17,18,19]. Therefore, introducing a small amount of RGO into the Bi_12_TiO_20_ microstructure could form an efficient path for charge carrier migration to improve the photocatalytic ability.

In this work, Bi_12_TiO_20_/RGO microstructures were fabricated through free-template and one-step hydrothermal synthesis by introducing reduced graphene oxide (RGO) on the polyhedral microstructure Bi_12_TiO_20_. The physical and optical properties of Bi_12_TiO_20_/RGO were analyzed by various characterizations. The effects of the reaction time and mineralizer on the formation of the Bi_12_TiO_20_ polyhedral microstructure were also investigated. The photocatalytic properties of Bi_12_TiO_20_/RGO for reducing Cr(VI) in the presence and absence of organic acid compounds were also discussed.

## 2. Material and Methods

### 2.1. Synthesis of Catalyst

The Bi_12_TiO_20_/RGO polyhedral microstructure was prepared by a hydrothermal method. Typically, a certain amount of graphene oxide (GO) was dispersed in 2 mol/L NaOH solution under sonication for 20 min, and then Bi_2_O_3_ and TiO_2_ with a mole ratio of [Bi]/[Ti] = 12:1 were added into the solution while it was continuously ultrasonically dispersed for 10 min. The theoretical mass ratio of Bi_12_TiO_20_ and GO was 1:0.01. After that, 80 mL of the mixture solution was transferred into a 100 mL Teflon-lined stainless-steel autoclave and hydrothermally reacted for 24 h at 220 °C. The obtained precipitate was washed several times until the pH attained neutral and the as-prepared sample was dried in an oven at 80 °C for 12 h. As references, the pure Bi_12_TiO_20_ was also prepared under the method similar to those above without added graphene oxide.

### 2.2. Characterization

Powder X-ray diffraction (XRD) patterns of the samples were characterized on a D8 Advance X-ray diffractometer (Bruker AXS, Karlsruhe, Germany) with Cu Kα radiation. The morphologies of the powders were analyzed by a Phenom Pro scanning electron microscope (Phenom Pro, Eindhoven, The Netherlands). The UV–vis spectra were measured by a Lambda 950 UV/vis/NIR spectrometer (Perkin Elmer, Waltham, MA, USA). The Fourier IR spectroscopy was performed by using a Nicolet-670 Fourier transform infrared spectrometer (ThermoFisher, Carlsbad, CA, USA).

### 2.3. Photocatalytic Activity

The photocatalytic activities of catalysts were evaluated under visible light Xenon lamp (PLS-SXE300(BF), Beijing, China) with a 400 nm cut-off filter (λ ≥ 400 nm). In a typical process, the Cr(VI) solution (50 μmol/L) was adjusted to pH 3.0 by nitric acid solution, and a 0.15 g sample was added to 100 mL Cr(VI) solution. To ensure the adsorption–desorption equilibrium between Cr(VI) and catalysts, the suspension was stirred in the dark for 120 min. After attaining equilibrium, a definite time interval under the illumination of visible light, the aliquots samples were taken out of the reaction suspensions. The reductive rate of Cr(VI) was detected by the diphenylcarbazide method at 540 nm with a UV-2550 UV–vis spectrophotometer. Further, to explore the effect of various organic acids on the Cr(VI) reduction, p-hydroxybenzoic acid (PHBA), chloroacetic acid, and citric acid, with a concentration of 0.1 μmol/L, were added to the Cr(VI) solution, respectively. 

## 3. Results and Discussion

### 3.1. Composition and Morphology

**Figure 1:**
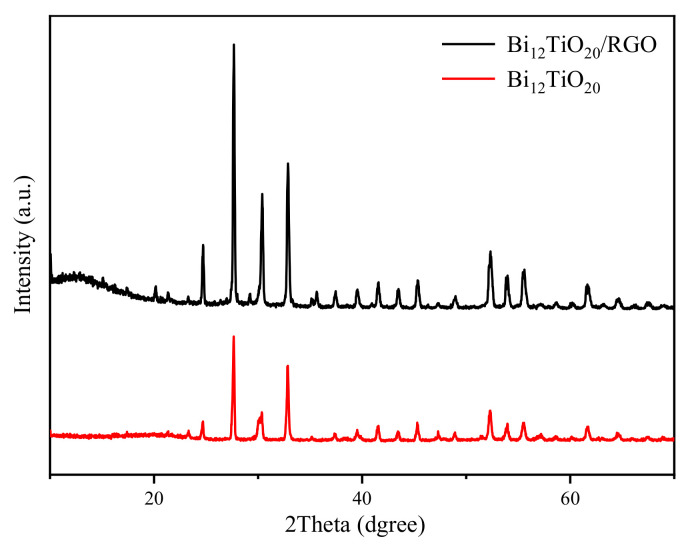
XRD patterns of the Bi_12_TiO_20_/RGO polyhedral microstructure and pure Bi_12_TiO_20_.

The phase and crystal structure of Bi_12_TiO_20_/RGO was analyzed by XRD. As shown in Figure 1, all diffraction peaks of the prepared Bi_12_TiO_20_/RGO samples could be well indexed to the cubic Bi_12_TiO_20_ (JCPDS No. 34-0097), which indicated that the crystal structure of Bi_12_TiO_20_ was stable for the fabrication of hybrid composites. The strong and sharp diffraction peaks indicated that Bi_12_TiO_20_ had good crystallinity in the composites. The XRD patterns for the RGO were not observed in Bi_12_TiO_20_/RGO, which is mainly because of the relatively low content level of RGO, as well as the RGO sheets being further exfoliated during hydrothermal treatment [8,20].

**Figure 2:**
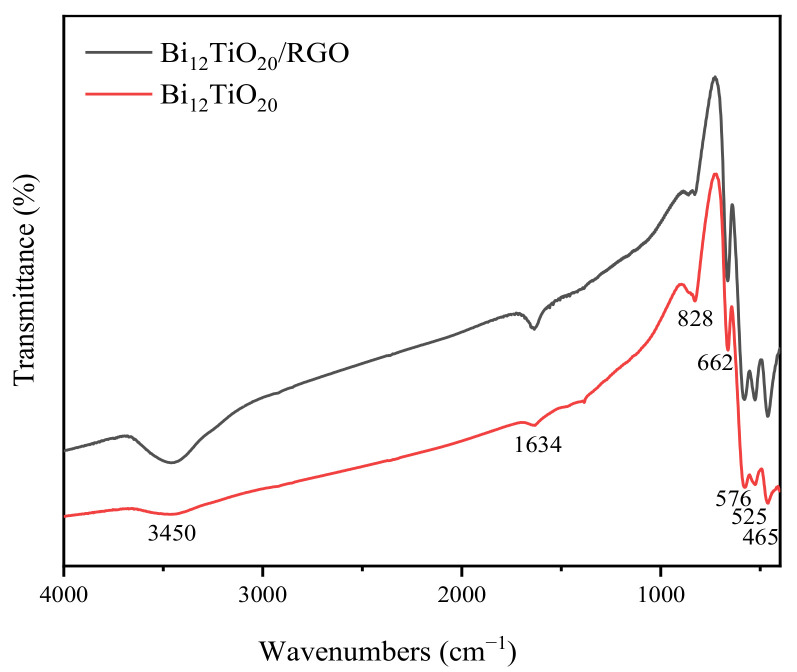
FT-IR spectra of Bi_12_TiO_20_/RGO and pure Bi_12_TiO_20_ Morphology analysis.

In order to detect the structure of the samples, Bi_12_TiO_20_ and Bi_12_TiO_20_/RGO were analyzed by FT-IR spectrum. As displayed in Figure 2, for pure Bi_12_TiO_20_, five peaks were shown at 459 cm^−1^, 524 cm^−1^, 576 cm^−1^, 662 cm^−1^, and 828 cm^−1^, respectively. These peaks all contributed to Bi–O vibration modes; this result was consistent with the characteristic peaks of sillenite. Because of the introduction of RGO, more peaks appeared in the Bi_12_TiO_20_/RGO samples. The peak located at 1634 cm^−1^ corresponded to stretching vibration modes of aromatic C=C bonds which are the basic unit of reduced graphene oxide. Moreover, the peak at 3450 cm^−1^ was attributed to hydroxyl (-OH) groups from the absorbed water and the residual of the reduced graphene oxide. Generally, these results revealed that Bi_12_TiO_20_ and Bi_12_TiO_20_/RGO were synthesized successfully.

**Figure 3:**
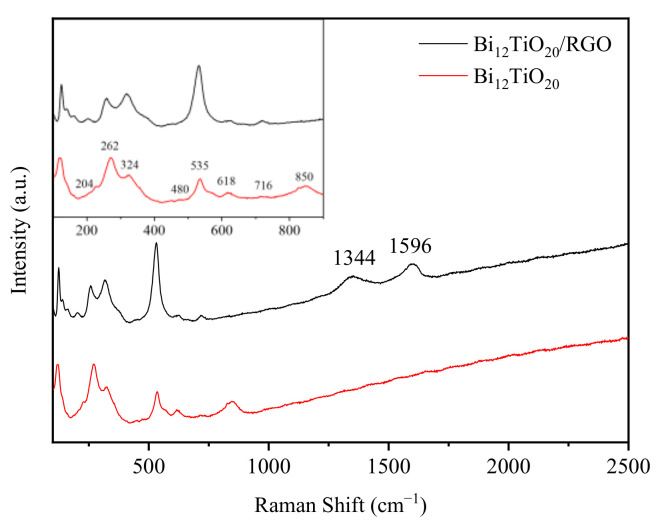
Raman spectra of Bi_12_TiO_20_/RGO and pure Bi_12_TiO_20_.

Figure 3 shows the Raman spectra of Bi_12_TiO_20_ and Bi_12_TiO_20_/RGO. The curves for Bi_12_TiO_20_ at 100 cm^−^^1^ versus 900 cm^−1^ are similar to previously reported data [21,22]. Specifically, the peaks at 204 and 262 cm^−^^1^ arose due to Bi-O bending and stretching vibrations and O-Ti-O bending vibrations, respectively. The peaks at 324 and 535 cm^−1^ were attributed to the O-O bonding vibrations in Bi_12_TiO_20_. The peaks at 716 and 850 cm^−1^ were attributed to the symmetric and antisymmetric stretching of the TiO_4_ tetrahedra, respectively [21,23].

The Bi_12_TiO_20_/RGO composite had essentially the same peaks as Bi_12_TiO_20_, except that it showed strong characteristic peaks in the Raman spectra at 1344 and 1596 cm^−^^1^, corresponding to the D and G bands of RGO, respectively [8,24], which indicated successful introduction of RGO. Both the D and G bands are characteristic peaks in the Raman spectra of different defects scattered by carbon atoms. The intensity ratio (ID/IG) of the D and G peaks can be used to assess the defect density of the carbon material [25,26]. A value of 0.89 for ID/IG in Bi_12_TiO_20_/RGO indicated the presence of exact crystal structure defects in Bi_12_TiO_20_/RGO [27], resulting in easier transport of photogenerated carriers in graphene and thus a lower probability of photoelectron-hole complexation.

**Figure 4:**
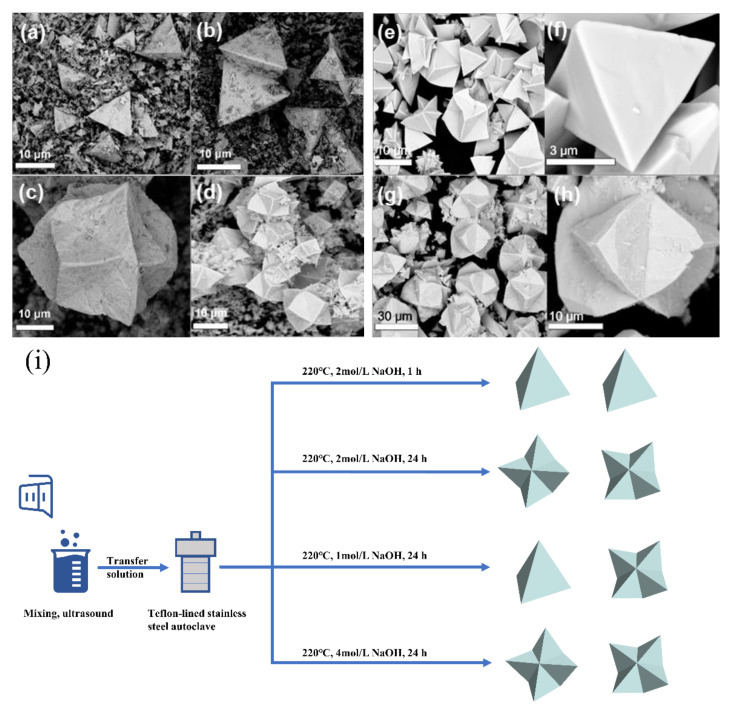
SEM images of the Bi_12_TiO_20_ synthesized at different times, 1 h (**a**), 2 h (**b**), 6 h (**c**), 24 h (**d**); and with different NaOH concentrations, 1 mol/L (**e**,**f**), 4 mol/L (**g**,**h**), Growth mechanism of Bi_12_TiO_20_ under different conditions (**i**).

**Figure 5:**
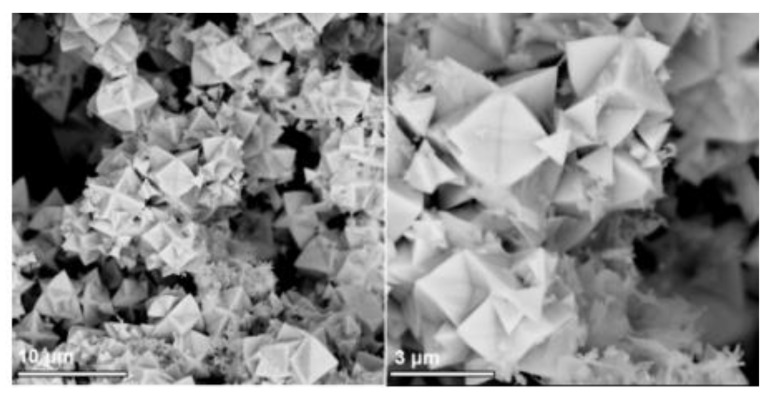
SEM images of the Bi_12_TiO_20_/RGO samples.

The morphologies of pure Bi_12_TiO_20_ at different hydrothermal times are observed in Figure 4a–d. It can be seen that, after the hydrothermal reaction for 1 h to 2 h, the Bi_12_TiO_20_ formed a mainly tetrahedral structure, and when the reaction time was increased to 6 h, the polyhedral microstructure appeared. With a 24 h reaction time, Bi_12_TiO_20_ mainly exited with a polyhedral microstructure. In addition to the hydrothermal time, the OH^−^ ions also played an important part in the morphology formation in the hydrothermal process [15,28]. As shown in Figure 4e,f, with 1 mol/L NaOH solution, the Bi_12_TiO_20_ showed a tetrahedral structure and non-uniform polyhedral microstructure. When the OH^−^ ions increased to 4 mol/L, the Bi_12_TiO_20_ can be described as a uniform polyhedral microstructure. According to relevant literature descriptions [15], the influence of OH^−^ anions on specific surfaces may affect the crystal growth direction and rate, which may be the cause of a uniform polyhedral microstructure. Therefore, the reaction time and the OH^−^ ions are key factors for controlling the development of the Bi_12_TiO_20_ polyhedral microstructure (Figure 4i). The morphologies of Bi_12_TiO_20_/RGO are shown in Figure 5. It can be seen that the Bi_12_TiO_20_/RGO still exhibited a polyhedral microstructure after the introduction of RGO and RGO had no effect on crystal surface growth. The small size and polyhedral microstructure will enhance light reflection and absorption, further contributing to the photocatalytic degradation effect. 

**Figure 6:**
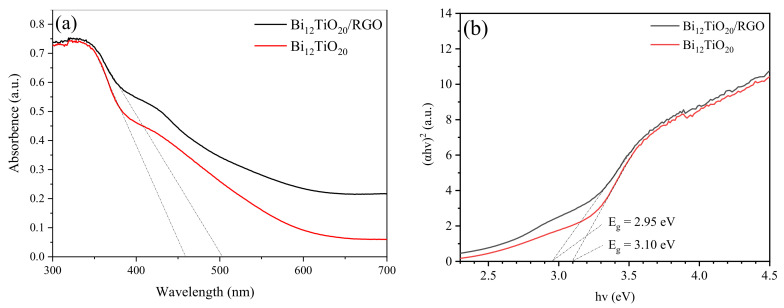
UV–vis diffuse reflectance spectra (**a**,**b**) Tauc plots of the Bi_12_TiO_20_/RGO and pure Bi_12_TiO_20_.

The UV–vis diffuse reflectance was conducted to evaluate the optical properties of each as-prepared sample. As shown in Figure 6a, pure Bi_12_TiO_20_ exhibited a photo-absorption from UV to visible light region, and its absorption onset was around 459 nm. Due to the background absorption of the reduced graphene, it was obvious that the absorbance intensities of Bi_12_TiO_20_/RGO strengthened in the range from 400 nm to 700 nm. On the other hand, there was a red shift in the absorbance onset from 459 to 503 nm due to the carbon modification of Bi_12_TiO_20_. As a result, the visible utilization of Bi_12_TiO_20_/RGO composites was enhanced. As shown in Figure 6b, After calculation [29], E_g_ values of Bi_12_TiO_20_/RGO and pure Bi_12_TiO_20_. were 2.95 eV and 3.1 eV, respectively. The E_g_ of the carbon-modified Bi_12_TiO_20_ became smaller, further demonstrating the enhanced utilization of visible light by Bi_12_TiO_20_/RGO.

### 3.2. Photocatalytic Reduction of Hexavalent Chromium under Visible Light

**Figure 7:**
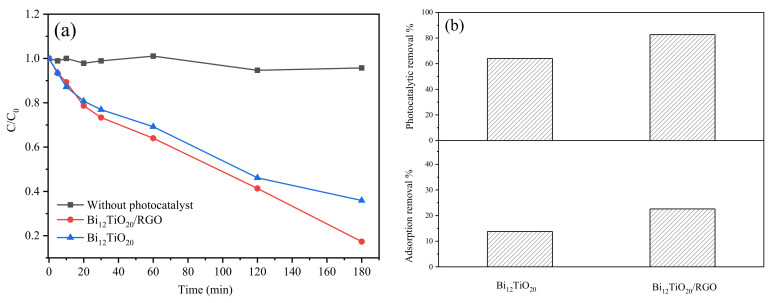
(**a**) C/C_0_ vs. time plot for the photocatalytic reduction of Cr(VI) by the Bi_12_TiO_20_/RGO and pure Bi_12_TiO_20_; (**b**) The photocatalytic removal and adsorption removal of Cr(VI) by the Bi_12_TiO_20_/RGO and pure Bi_12_TiO_20_.

The photocatalytic reduction ability of Cr(VI) using Bi_12_TiO_20_ and Bi_12_TiO_20_/RGO were evaluated. Before irradiation, the adsorption of Cr(VI) in solution of the samples was in equilibrium after 120 min, and the adsorption removal was 13.8% and 22.6% for pure Bi_12_TiO_20_ and Bi_12_TiO_20_/RGO, respectively (In Figure 7b). Bi_12_TiO_20_/RGO showed better adsorption for the removal of Cr(VI) than pure Bi_12_TiO_20_. As shown in Figure 7, within 180 min visible light irradiation, the removal rate of Cr(VI) by pure Bi_12_TiO_20_ was just 64.1%, while the removal rate of the Bi_12_TiO_20_/RGO reached 82.7%. Additionally, according to the equation of the pseudo-first-order kinetics model: −*ln*(*C/C*_0_) = *kt*, where *C*_0_ is the initial concentration for each pollutant, *C* is the instantaneous concentration at the irradiating time of *t*, and *k* is the first-order rate constant for degradation. The corresponding reaction rate constants were calculated to be 0.0085 min^−1^ and 0.0060 min^−1^ for Bi_12_TiO_20_/RGO and Bi_12_TiO_20_, respectively. There is some literature on the reduction removal of Cr(VI) by designing a photocatalysis system and limited reports using Bi_12_TiO_20_ photocatalyst. The TiO_2_-WO_3_ incorporated PANI membrane showed 98.50% removal of Cr(VI) by filtration and 67.32% by photocatalytic reduction [9]. The Biochar/Bi/Fe_3_O_4_ composites presented 95% adsorption-photocatalytic removal for Cr(VI) in 180 min under visible light irradiation [30]. By using the graphene-based composite ((BOCI/CNCl/rGH), the removal efficiencies reached 87% and 85% in a tetracycline hydrochloride and Cr(VI) coexistence system, higher than those in a TCH or Cr(VI) alone system [31]. Compared with the reported literature, the Bi_12_TiO_20_/RGO polyhedral microstructure exhibited great potential for the visible-light photoreduction removal system of Cr(VI). With the introduction of RGO, the Bi_12_TiO_20_/RGO exhibited both better adsorptive and photocatalytic ability.

**Figure 8:**
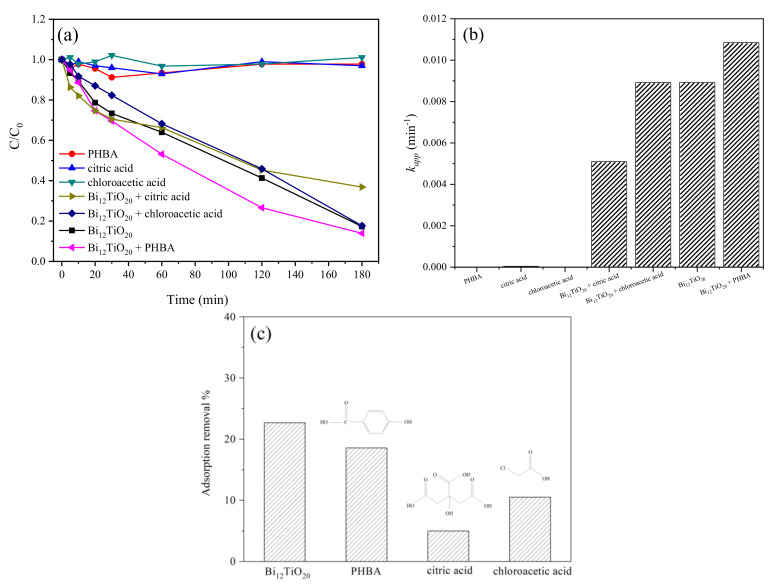
(**a**) *C*/*C*_0_ vs. time plot and (**b**) *k_app_* for the photocatalytic reduction of Cr(VI) by Bi_12_TiO_20_/RGO with the addition of PHBA, chloroacetic acid, and citric acid, respectively; (**c**) The adsorption removal of Cr(VI) by the Bi_12_TiO_20_/RGO with PHBA, chloroacetic acid, and citric acid, respectively.

Furthermore, different organic acids, PHBA, chloroacetic acid, and citric acid were added to explore the photocatalytic reduction of Cr(VI) by Bi_12_TiO_20_/RGO. Generally speaking, the addition of organics are beneficial for the photocatalytic reduction of Cr(VI) by improving the separation of photogenerated charge via the scavenging of holes in the photocatalytic reactions [32,33]. As shown in Figure 8a,b, with the addition of PHBA, there was an obvious acceleration of the Cr(VI) photocatalytic removal. Under 180 min visible light irradiation with the addition of PHBA, the degradation rate increased by 3.4% and the kinetic constant increased from 0.0089 min^−1^ to 0.011 min^−1^. While for Cr(VI), the photocatalytic removal was not obviously improved with the addition of chloroacetic acid, and even decrease with citric acid, and the kinetic constant decreased from 0.0089 min^−1^ to 0.0051 min^−1^. It might be because of the competitive adsorption between organic acid and Cr(VI). From Figure 8c, the Cr(VI) adsorption depressed with the addition of PHBA, chloroacetic acid, and citric acid, and the adsorption removal was significantly decreased with citric acid. The result indicated that the Cr(VI) adsorption could largely influence the photocatalytic reduction reaction in the Cr(VI) and organic acid combined pollutants.

### 3.3. Catalytic Mechanism

**Figure 9:**
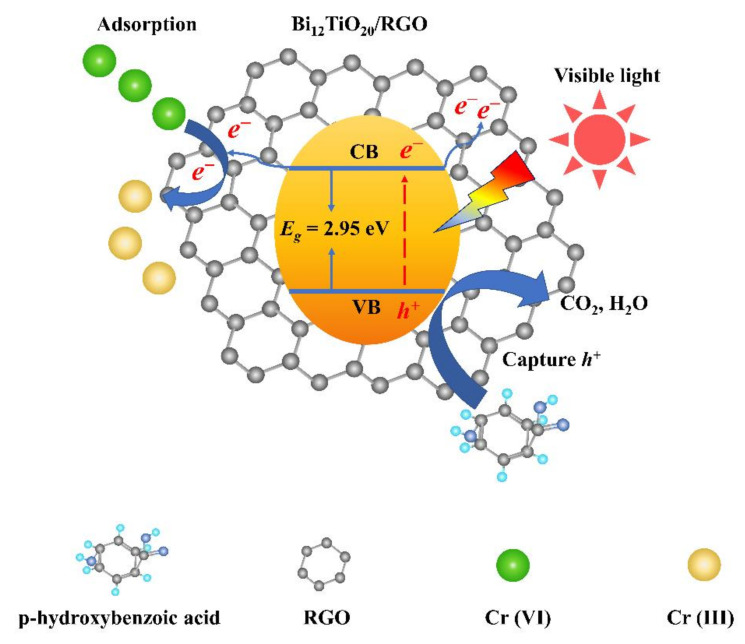
Mechanism of photocatalytic reduction of Cr (VI) in Bi_12_TiO_20_/RGO organic acid system.

A possible mechanism for the photocatalytic reduction of Cr(VI) by Bi_12_TiO_20_/RGO polyhedral microstructure composites can be interpreted. Generally, an increased adsorption ability for Cr(VI), better light absorbing quality, and efficient photoexcited charge transfer could be the significant factor for the enhanced photocatalytic activity of Bi_12_TiO_20_/RGO. Under the driving force of electrostatic attraction, Cr(VI) is abundantly concentrated on the surface of the Bi_12_TiO_20_/RGO polyhedral microstructure and adsorbed on the surface of catalyst particles. It is well known that the photocatalytic reduction of Cr(VI) is a heterogeneous reaction, and the enrichment of Cr(VI) onto the photocatalyst has a great influence on the photocatalytic performance improvement. Moreover, according to the result of UV–vis spectra, the introduction of RGO could improve the utilization of visible light, and a better absorption quality is beneficial to the development of photocatalytic activities. The photoelectrons and hole separation are also critical factors. The mechanism of Cr (VI) reduction in the Bi_12_TiO_20_/RGO system is shown in Figure 9. Under visible light irradiation, photoexcited electrons in the valence band of Bi_12_TiO_20_ may transfer to the conduction band due to the internal electric field. RGO is a great electron acceptor to reserve photoexcited electrons [34,35]. It hinders the recombination of photoexcited charges and prolongs their lifetime. The transferring electrons reserved on the RGO also could be trapped by Cr(VI) ions adsorbed on the surface of Bi_12_TiO_20_/RGO, and thus the Cr(VI) is reduced to Cr(III). The photogenerated holes may react with the adsorbed water on the surface to form oxygen and hydrogen ions. It may also be oxidized to hydroxyl radicals •OH [36,37], which could oxidize Cr(III) backward to Cr(VI), and the photocatalytic reduction of Cr(VI) efficiency of the photocatalyst could be suppressed. With the addition of p-hydroxybenzoic acid, photogenerated holes could be trapped by p-hydroxybenzoic acid and considerably promote the charge separation, resulting in the improved photocatalytic reduction of Cr(VI) on the surface of Bi_12_TiO_20_/RGO. However, with the addition of chloroacetic acid and citric acid, the Cr(VI) photocatalytic reduction is not expected to be promoted. The competitive adsorption of chloroacetic acid and citric acid with Cr(VI) might be the dominant reason for this result. Many activity sites at catalysts were occupied by the organic acid, and photoexcited electrons could also be adsorbed hunted by O_2_ to form O_2_^•−^ radicals, so the charge separation effect of the organic acids (chloroacetic acid and citric acid) did not obviously result in promoting Cr(VI) photocatalytic reduction, and even depressed the Cr(VI) reduction.

## 4. Conclusions

A Bi_12_TiO_20_/RGO polyhedral microstructure was synthesized using a simple one-step hydrothermal process. The RGO nanostructures were introduced to the Bi_12_TiO_20_ microstructures from the XRD and FT-IR characterization. The SEM observations indicated that reaction time and the OH^−^ ions concentration played an important role in the formation of Bi_12_TiO_20_ miro-polyhedral morphology. The improved photocatalytic properties of the Bi_12_TiO_20_/RGO for the reduction of Cr(VI) was mainly attributed to the better photogenerated electrons transfer using RGO as a promoter. Moreover, better adsorption and enrichment of Cr(VI) onto the composite catalysts provide more active sites for the photocatalytic reaction. In the present of organic acids, the promotion effects of the organic acids on Cr(VI) photocatalytic reduction were limited by the competitive adsorption between the organics and Cr(VI). This work verified that microstructure control was an efficient method to improve the photoreduction activity of Bi_12_TiO_20_-based photocatalysts. In the future, further tests will be broadened on the long-term photoreduction test of hexavalent chromium using Bi_12_TiO_20_-based membranes, with the aim of potential application.

## Data Availability

The data is available on reasonable request from the corresponding author.
